# Metapangenomic investigation provides insight into niche differentiation of methanogenic populations from the subsurface serpentinizing environment, Samail Ophiolite, Oman

**DOI:** 10.3389/fmicb.2023.1205558

**Published:** 2023-07-03

**Authors:** Patrick H. Thieringer, Eric S. Boyd, Alexis S. Templeton, John R. Spear

**Affiliations:** ^1^Department of Civil and Environmental Engineering, Colorado School of Mines, Golden, CO, United States; ^2^Department of Microbiology and Cell Biology, Montana State University, Bozeman, MT, United States; ^3^Department of Geological Sciences, University of Colorado, Boulder, CO, United States

**Keywords:** serpentinization, subsurface, pangenomics, niche differentiation, geomicrobiology

## Abstract

Serpentinization reactions produce highly reduced waters that have hyperalkaline pH and that can have high concentrations of H_2_ and CH_4_. Putatively autotrophic methanogenic archaea have been identified in the subsurface waters of the Samail Ophiolite, Sultanate of Oman, though the strategies to overcome hyperalkaline pH and dissolved inorganic carbon limitation remain to be fully understood. Here, we recovered metagenome assembled genomes (MAGs) and applied a metapangenomic approach to three different *Methanobacterium* populations to assess habitat-specific functional gene distribution. A Type I population was identified in the fluids with neutral pH, while a Type II and “Mixed” population were identified in the most hyperalkaline fluids (pH 11.63). The core genome of all *Methanobacterium* populations highlighted potential DNA scavenging techniques to overcome phosphate or nitrogen limitation induced by environmental conditions. With particular emphasis on the Mixed and Type II population found in the most hyperalkaline fluids, the accessory genomes unique to each population reflected adaptation mechanisms suggesting lifestyles that minimize niche overlap. In addition to previously reported metabolic capability to utilize formate as an electron donor and generate intracellular CO_2_, the Type II population possessed genes relevant to defense against antimicrobials and assimilating potential osmoprotectants to provide cellular stability. The accessory genome of the Mixed population was enriched in genes for multiple glycosyltransferases suggesting reduced energetic costs by adhering to mineral surfaces or to other microorganisms, and fostering a non-motile lifestyle. These results highlight the niche differentiation of distinct *Methanobacterium* populations to circumvent the challenges of serpentinization impacted fluids through coexistence strategies, supporting our ability to understand controls on methanogenic lifestyles and adaptations within the serpentinizing subsurface fluids of the Samail Ophiolite.

## Introduction

Earth’s deep subsurface biosphere requires substrates created from water-rock interactions to support a continued source of chemical energy for microbial metabolisms. Serpentinization—the hydration and oxidation of ultramafic, olivine-rich rock connected to the reduction of water—produces the secondary minerals serpentine, iron oxides, as well as hydroxides, and molecular hydrogen (H_2_; Sleep et al., 2004; Schulte et al., 2006; McCollom and Seewald, 2013). Surface derived inorganic carbon (CO_2_) can be abiotically reduced to methane (CH_4_) and other potential hydrocarbons or organic acids (Holm and Charlou, 2001; McCollom and Seewald, 2013; Miller et al., 2017). These water-rock reactions produce fluids that are geochemically reduced with very low oxidation–reduction potentials. These serpentinized-impacted waters generate conditions of alkaline to hyperalkaline pH values from 8 to greater than 12, while producing appreciable concentrations of H_2_ and CH_4_ that can readily serve as electron donors for subsurface microbial metabolisms (Russell et al., 2010; Brazelton et al., 2012; Schrenk et al., 2013; Miller et al., 2016; Suzuki et al., 2017). Serpentinization acts as a viable process for supporting chemolithoautotrophic life in the deep subsurface through abiotic reactions.

Sites of terrestrial serpentinization are distributed globally and show evidence of methanogenic and methanotrophic microbial members, including the Voltri Massif (Italy), Santa Elena Ophiolite (Costa Rica), the Chimaera Seeps (Turkey), and the Manleluag Spring National Park of Zamabales (Philippines; Brazelton et al., 2017; Crespo-Medina et al., 2017; Zwicker et al., 2018; Wang et al., 2022). Specifically, the Samail Ophiolite, Sultanate of Oman, offers the largest exposed ophiolite on Earth that is actively undergoing low temperature serpentinization (Neal and Stanger, 1985; Kelemen and Matter, 2008; Kelemen et al., 2011; Paukert et al., 2012; Miller et al., 2016). Recent work has determined the presence of an active CH_4_ cycle in the subsurface fluids of the Samail Ophiolite, where methanogenic populations are most abundant and transcriptionally active in the hyperalkaline fluids (Kraus, 2021). Additional ^14^C-labeled substrate microcosm assays confirmed the generation of CH_4_ and assimilation of organic substrate into biomass from organisms within the subsurface fluids (Fones et al., 2019). Investigation into the methanogenic community has revealed the diversification of the genus *Methanobacterium*, reflecting species inhabiting the hyperalkaline fluids capable of potentially utilizing formate as an alternative carbon source and electron donor (Fones et al., 2021). These incubation studies have provided initial insights regarding the carbon metabolic capabilities of *Methanobacterium*, yet further investigation is required to understand the unique pH and nutrient limitation strategies employed by *Methanobacterium* populations to overcome environmental stressors controlling methanogenic activity in subsurface serpentinizing conditions.

The genomic relatedness of a group of organisms can be studied with the use of pangenomics to contrast population diversity and functional capabilities (Medini et al., 2005; Tettelin et al., 2005; Vernikos et al., 2015). The pangenome can identify the core genome shared across an entire group, as well as the accessory genome that is specific to individual members or sub-groups (Delmont and Eren, 2018). The taxonomic resolution of pangenomics can be applied at higher order rankings in order to demonstrate the relatedness of members and provide informative comparisons across phylogenetic or environmental associations (Simon et al., 2017; Utter et al., 2020). However, the application of pangenomics to environmental systems remains understudied. Pangenomic analyses have primarily focused on genomes of cultivars to describe the pangenome of a well-studied taxon. Contrastingly, pangenomic tools applied to organisms difficult to cultivate from the environment can reveal the genetic potential across a taxon of interest and the various sub-populations separated by environmental conditions. The combination of metagenomics and pangenomics allows for the opportunity to investigate microbial members across complex environmental conditions (Simmons et al., 2008; Kashtan et al., 2014). Sampling for metagenomes can provide information of microbial populations within their environmental setting, and pangenomic investigation can reveal the biogeography of microbial diversity and gene distribution (Delmont and Eren, 2018). This combination of tools termed “metapangenomics” can inform on adaptability to environmental conditions, and broadly investigate the evolution of microbial members across diverse habitats (Delmont and Eren, 2018). The Samail Ophiolite provides an ideal environmental system with distinct geochemical conditions across fluid types to investigate the adaptability of microbial populations within hyperalkaline settings. Methanogen populations serve as a prominent target for metapangenomic evaluation due to their abundance across pH and geochemical conditions to understand their biogeography and habitat-specific gene pools.

While preliminary work has highlighted some metabolic adaptations that enable *Methanobacterium* to function in the subsurface fluids of the Samail Ophiolite (Fones et al., 2019, 2021), further efforts to characterize how *Methanobacterium* overcome the polyextremic conditions in the hyperalkaline wells and maintain such a dominating presence are needed. Here we apply metagenomics to examine how *Methanobacterium* populations potentially overcome DIC limited and hyperalkaline pH conditions. A metapangenomic analysis was used to describe the adaptations of *Methanobacterium* populations spanning multiple subsurface wells at various pH conditions and depth profiles to highlight adaptive strategies through carbon utilization, use of transporters, and other survival mechanisms. The work herein helps to reveal how *Methanobacterium* populations exhibit niche differentiation for cohabitation and circumvent the energy limiting conditions induced from low temperature serpentinization in the subsurface fluids of the Samail Ophiolite.

## Materials and methods

### Site description and geochemical measurements

Three preexisting boreholes were sampled from a multi-borehole observatory established by the Oman Drilling Project and Oman Ministry of Regional Municipalities and Water Resources in Wadi Lawayni in the Wadi Tayin massif into the mantle section of the Samail Ophiolite (Kelemen et al., 2011; Templeton et al., 2021). Fluid samples were collected in February to March of 2020 using multiple devices to cover a range of depth intervals within each borehole. A Grundfos SQ 2–85 submersible pump (Grundfos Pumps Corp. Denmark) was used for “open” borehole pumping. This pumping method was conducted at 50 m in borehole WAB188 and 75 m in borehole BA3A. A Double Packer Standard System (SolExperts, France) was used in borehole NSHQ14. The packer system contains two inflatable rubber packers and a submersible pump, and one or both of the packers can be inflated to isolate depth intervals for discrete pumping of fluids as described in previous work (Nothaft et al., 2021a). The top packer was left uninflated to sample from the top of the water table (9 m) to 30 m where the bottom packer was set. The submersible pump and packer system were connected to a splitting manifold with field-washed Tygon tubing. An air-tight gas sampler was used in borehole BA3A by dropping at 100 and 275 m depth, capturing a ~1 m interval of approximately 5 L of fluid.

Prior to sampling, a field wash lasting ~20–30 min was performed on the pump, manifold, tubing and filter housing at each borehole. Fluids were collected for geochemical analysis by passing through a 0.2 μm polycarbonate filter into 15 mL Falcon tubes. Major cations were acidified with nitric acid in the field at the time of collection. As previously reported (Kraus, 2021), anion and cation concentrations were analyzed using inductively coupled plasma atomic emission spectroscopy (ICP-AES; Optima 5300, Perkin-Elmer, Freemont, CA) and ion chromatography (IC; ICS-90, Dionex, Sunnyville, CA) respectively at the Colorado School of Mines. Water temperature and pH were measured in the field with a Hach multiparameter field meter (HQ40D, Hach, Inc., Loveland, CO).

### Sample collection, DNA extraction, and metagenomic sequencing

Biomass was collected from fluids passed through 0.2 μm polycarbonate filters when sampling from the submersible pump or packer system, or 0.1 μm filters from the air tight gas sampler. Approximately 5 L of fluid was passed through each filter or until noticeable particulates were collecting on the filter to ensure enough biomass was retrieved; in the case of the 0.1 μm filters, a minimum of 200 mL was passed through or until it was too difficult to deliver any liquid through the filter. Filters were immediately stored in DNA/RNA Shield™ (Zymo, Inc.) to be preserved until returned to the lab. DNA and RNA were extracted using the ZymoBIOMICS™ DNA/RNA Miniprep kit as described in previous work (Thieringer et al., 2021). Five samples representing each sampling depth from the boreholes described above were submitted for metagenomic sequencing on an Illumina NovaSeq platform (2 × 150 bp) and processed as a part of the Joint Genome Institute Pipeline, described in detail previously (Clum et al., 2021).

Metagenomic sequences were downloaded from the JGI/IMG portal, which already included steps of read quality filtering and trimming of adapters using BBduk (Bushnell, 2020). Reads were individually assembled using SPAdes with the “—meta” option for metagenomic data (Nurk et al., 2017). Individual reads were then mapped to contigs within each respective individual metagenome sample using Bowtie2 to generate coverage data (Langmead and Salzberg, 2012). Open reading frames (ORFs) were identified on the contigs with the program Prodigal and generated into contigs database files for downstream analysis with the program Anvi’o (Hyatt et al., 2010; Eren et al., 2015). Functional annotation of ORFs were identified with HMMER, Clusters of Orthologous Genes (COGS), Pfam, Kegg (through GhostKOALA), and with Interproscan using TIGRFAM and SUPERFAMILY (Zhang and Wood, 2003; Bateman, 2004; Kanehisa et al., 2007; Mulder and Apweiler, 2007; Haft et al., 2012; Galperin et al., 2021). Initial taxonomic annotation was provided by running the “anvi-estimate-scg-taxonomy” referencing the GTDB database, and helped guide binning efforts at the refinement stage (Eren et al., 2015). The contigs databases and BAM files created during the Bowtie2 mapping step were then created into a profile database within Anvi’o (Langmead and Salzberg, 2012; Eren et al., 2015). Initial binning was performed with CONCOCT, Maxbin2, and MetaBat2 software packages using default parameters (Alneberg et al., 2013; Wu et al., 2016; Kang et al., 2019). Bins were then integrated into DASTool to calculate the most optimized and non-redundant set of bins from each metagenome (Sieber et al., 2018). These binning results were imported into the profile database and then manually refined into metagenome assembled genomes (MAGs) within the Anvi’o interactive interface (Eren et al., 2015). MAGs were considered high-quality based upon >90% completion and <10% redundancy thresholds (Eren et al., 2015).

Phylogenomic analyses of *Methanobacterium* MAGs were conducted by using the program GtoTree (Lee, 2019). In brief, representative *Methanobacterium* genomes were downloaded as accession files from the Genome Taxonomy Database (GTDB), where only species cluster representatives were selected using the “—GTDB-representatives-only” flag. An outgroup genome representative was downloaded from NCBI for phylogenetic reconstruction, which included a genome for *Methanosarcina barkeri*. GtoTree was run with default settings except for the input single copy gene HMM sets using the “-H” flag, and the archaeal HMM target gene set was used for analysis which contains 76 marker genes. GtoTree then identifies target genes from MAGs and genomes using HMMER3, aligns each gene set with the MUSCLE program, and performs automated trimming with TrimAl (Zhang and Wood, 2003; Edgar, 2004; Capella-Gutierrez et al., 2009). The alignment and partitions files were then passed on to IQTree where a maximum likelihood phylogenetic analysis was conducted by identifying the optimal amino acid substitution model by implementing the “-m TEST” flag, and branch support was conducted with 1,000 ultrafast bootstraps (Minh et al., 2020). Tree files were then visualized and annotated with the Interactive Tree of Life (iTOL) web program (Letunic and Bork, 2007).

### Metapangenomic workflow

Eight MAGs taxonomically identified as *Methanobacterium* considered high quality, from completion and redundancy scores, were examined through the pangenomic workflow outlined by Anvi’o (Eren et al., 2015; Utter et al., 2020). In brief, the workflow computes the amino acid identity level between all ORF pairs using BLASTp, and removes matches below a bitscore (at default value of 0.5; Altschul et al., 1990). Homologous gene clusters were grouped from ORFs using the Markov Clustering Algorithm (MCL; Eren et al., 2015; Utter et al., 2020). These gene clusters were then aligned using MUSCLE for interactive visualization (Edgar, 2004). Core genes and gene clusters are identified as sharing at least a fourth of the median coverage of where the sample gene originated from, otherwise the gene or gene cluster was determined to be accessory as described previously (Eren et al., 2015; Utter et al., 2020). The core and accessory genomes of groups were binned according to overlapping gene clusters identified in the Anvi’o interactive interface. Core and accessory genomic information determined from a metapangenomic approach does not differ from pangenomic analysis in terms of its meaning. Instead, a metapangenome incorporates metagenomic read recruitment to aid in identifying core and accessory genomic content across environmental conditions. The average nucleotide identity (ANI) of each MAG was calculated with the function “anvi-compute-genome-similarity” which calls the PyANI program (Pritchard et al., 2016). The “ANI_full_percent_identity” option was included in the final pangenome figure and used to compare the genome similarity of the eight *Methanobacterium* MAGs. This option takes into account both the percent identity of the aligned fraction from the MAGs being compared, as well as the aligned fraction or coverage of the MAGs. Inclusion of coverage/alignment fraction provides greater stress on the likelihood of the MAGs representing different species due to greater homology shared from greater alignment fractions (Pritchard et al., 2016). The command “anvi-summarize” was run on the pangenome and contigs databases to export the genes and gene clusters within each pangenome grouping (accessory or core). The exported text file was then curated in R and visualized with custom R scripts with the “ggplot2” package (Wickham, 2011; R Core Team, 2021).

A functional enrichment analysis was conducted on the eight MAGs assembled from this study in order to highlight gene clusters unique to the Oman *Methanobacterium* populations as a whole. Publicly available genomes (*n* = 57) for *Methanobacterium* were downloaded from the NCBI Genbank and Refseq databases, and the two other MAGs from wells NSHQ14 and WAB188, to contrast genes that may be unique to the MAGs from these hyperalkaline conditions within the Samail Ophiolite. In brief, a pangenome was conducted as above, this time including the MAGs identified in this study and external genomes (Supplementary Figure 1). The function “anvi-compute-functional-enrichment-in-pan” was run in order to identify which gene clusters are enriched a within the Oman *Methanobacterium* MAGs. Additional enrichment analyses were conducted on each *Methanobacterium* population against the others in order to compare and contrast genes that may serve as functionally core within each population type.

## Results

### Geochemical characterization of subsurface fluids

Subsurface fluids were sampled from three preexisting wells in the Samail Ophiolite for molecular biological and chemical analysis. The waters are classified based on previously reported data to reflect the geologic and hydrologic conditions of the fluids from each borehole—this includes Type I and Type II depending on the pH of the water and concentrations of Mg^2+^ and HCO_3_^−^ or Ca^2+^ and OH^−^ (Rempfert et al., 2017). Hyperalkaline fluids retrieved from BA3A and NSHQ14 agree with previous classification, with pH values of 11.63 and 11.24, respectively, and are reflective of Type II waters resembling closed system—no exposure to atmospheric input—serpentinization. Samples collected from WAB188 represents a “contact” zone where faulted boundary between gabbro and peridotite bedrock exists and a neutral pH of 7.47 was measured and the fluid composition reflect Type I characterizations of open system serpentinization. DIC is typically in very small concentrations (~0.05–0.13 mM) in the hyperalkaline fluids of NSHQ14 and BA3A, and greater concentrations in the circumneutral fluids of WAB188 (~3 mM; Rempfert et al., 2017; Fones et al., 2021; Nothaft et al., 2021b). Other potential sources of carbon generated as a result of serpentinization include acetate and formate, which were present in variable concentration in all fluids sampled. Acetate concentrations within hyperalkaline fluids were nearly double the concentrations of formate (1.01–2.48 μM acetate and 0.53–1.24 μM formate), while both analytes remained nearly equal in the neutral fluids (1.59 μM acetate and 1.63 μM formate). NO_3_^−^ concentrations are variable across fluid types. Nitrate was not detected in well BA3A, while measured in low concentrations (3.23 × 10^−3^ mM) within NSHQ14. Conversely, NO_3_^−^ concentrations in WAB188 were observed at 0.13 mM. NH_4_^+^ was not measured for well WAB188, however, was detected in large concentrations within the hyperalkaline fluids of NSHQ14 and BA3A (7.59–88.81 μM). Measurements for phosphorous species were not resolved and have not been reported in recent work conducted at the Samail Ophiolite. Further details of geochemical measurements from the 2020 field sampling campaign are reported in Table 1.

**Table 1:** Select geochemical compositions from subsurface waters recovered from wells BA3A, NSHQ14, and WAB188 collected from the Samail Ophiolite in 2020.

	WAB188	BA3A	NSHQ14	LOQ
Depth (m)	50	75	9–30	
pH	7.47	11.63	11.35	
Eh (mV)	–	–403	–169	
Temp. (°C)	35.3	36.8	35.9	
H_2_ (μM)	0.92	–	21–164	0.45
DIC (mM)	3	–	0.05–0.13	0.1
Acetate (μM)	1.59	2.48	1.01	0.07
Formate (μM)	1.63	1.24	0.53	0.24
SO_4_^2−^ (mM)	1.04	0.01	0.13	1.04 × 10^−3^
∑Na (mM)	2.73	9.94	6.84	1.25 × 10^−3^
∑Ca (mM)	1.21	6.2	3.35	1.22 × 10^−4^
∑Mg (mM)	1.7	0.03	0.03	1.62 × 10^−3^
NO_3_^−^ (mM)	0.15	–	3.23 × 10^−3^	1.61 × 10^−3^
NH_4_^+^ (μM)	–	88.81	7.59	1
∑Fe (μM)	13.61	6.26	2.33	5.37 × 10^−6^
∑Ni (μM)	0.14	0.1	0.14	2.39 × 10^−5^

A dashed line (−) indicates the sample was not measured. The concentrations of H_2_ and DIC were not possible and previous measurements from the 2015/2016 field season are reported from Rempfert et al. (2017) instead.

### Description of recovered MAGs

**Figure 1:**
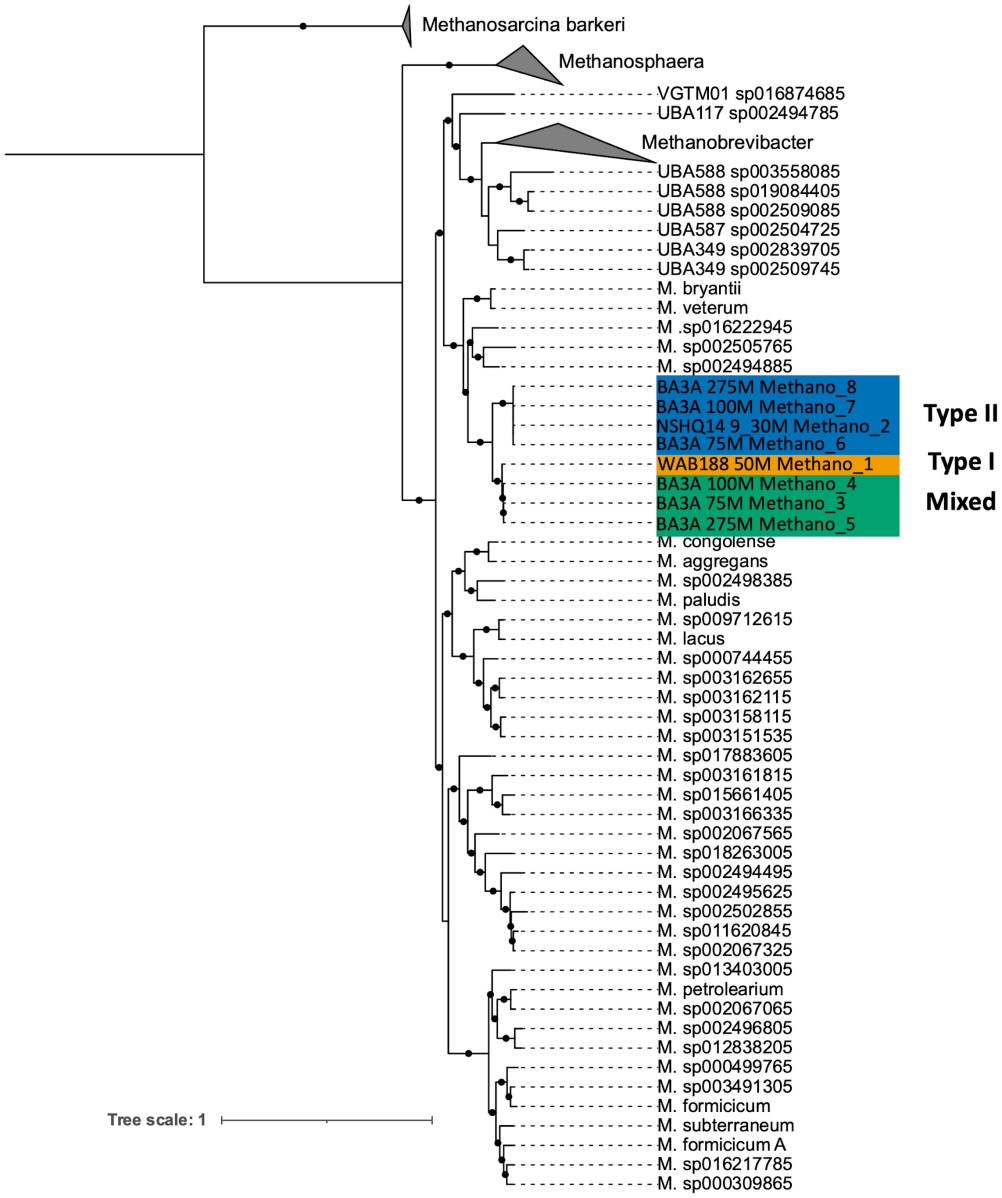
Maximum likelihood phylogenetic reconstruction of *Methanobacterium* metagenome assembled genomes based on 76 archaeal marker genes (Lee, 2019). The well and depth from which each MAG was collected is denoted in the sample name, along with the MAG ID. The MAGs are color-coded based on their population designation: blue = type II, green = mixed, yellow = type I. Bootstrap values ≥ 90% out of 1,000 bootstraps are denoted with black circles. *Methanosarcina barkeri* representatives were chosen as the outgroup.

DNA collected from filtered biomass for metagenomic analysis provided eight high quality MAGs representative of *Methanobacterium* populations. One MAG was recovered from WAB188 at 50 m depth, and another at the shallow packer interval of 9–30 m from NSHQ14. The six remaining MAGs were all recovered from well BA3A where two MAGs were identified at each sampling depth (75, 100, and 275 m). Phylogenomic analysis revealed the presence of three distinct populations of the *Methanobacterium* genus from subsurface fluids of the Samail Ophiolite. *Methanobacterium* populations are referred to by previous classification schemes reflecting the lithologic and hydrologic conditions reflected at the site of each MAG; Type I populations are indicative of alkaline conditions from near-surface water/rock interaction, whereas Type II populations reflect the highly serpentinized, closed-system conditions within hyperalkaline fluids in peridotite bedrock. The MAG from WAB188 represents the Type I population, while a distinct clade of 4 MAGs from BA3A and NSHQ14 represent the Type II population. Interestingly, the third population consisted of 3 MAGs and appeared to have recently diverged from the WAB188 Type I population and are phylogenetically distinct (Figure 1). This unique population shares the most phylogenetic relatedness to the Type I lineage, yet exists only in the most hyperalkaline well (BA3A, 11.63 pH). Therefore, this distinct population will hereafter be referred to as the “Mixed” population in order to distinctly evaluate the three populations throughout the remainder of this study. Comparatively, the Type II *Methanobacterium* MAGs exhibit the smallest genome size, while the Type I MAG contains the largest genomes size and Mixed population MAGs are slightly smaller than the Type I population (Table 2). This is consistent with previously observed genomes in other sites impacted by serpentinization, where genome streamlining allows for the reduction of energy demands within increasingly hyperalkaline conditions (Suzuki et al., 2017; Fones et al., 2019, 2021). While the MAGs from WAB188 and NSHQ14 only represent one sampling depth, the estimated relative abundance of the Mixed and Type II populations appear to increase with depth within well BA3A (Table 2).

**Table 2:** The statistics and information of assembling *Methanobacterium* MAGs recovered from the subsurface fluids.

MAG ID	Well	Depth (m)	Type	Est. comp. (%)	Est. red. (%)	Est. rel. abund. (%)	GC content (%)	Genome size (Mbp)
***Methanobacterium***								
Methano_1	WAB188	50	I	98.68	1.32	4.04	36.63	2.47
Methano_2	NSHQ14	9–30	II	98.67	0	5.03	34.76	1.64
Methano_3	BA3A	75	Mixed	98.62	1.31	1.67	36.64	2.07
Methano_4	BA3A	100	Mixed	98.68	1.32	7.57	34.15	2.12
Methano_5	BA3A	275	Mixed	94.76	2.63	7.91	34.59	2.29
Methano_6	BA3A	75	II	98.68	0	12.17	34.75	1.37
Methano_7	BA3A	100	II	98.67	0	13.08	35.03	1.59
Methano_8	BA3A	275	II	97.36	0	23.87	34.93	1.38

The relative abundance of each MAG was estimated by the percentage of overall reads mapped to a MAG within each of their corresponding metagenome. Est. comp., estimated completion; Est. red., estimated redundancy; Est. rel. abund., estimated relative abundance.

### *Methanobacterium* pangenome

**Figure 2:**
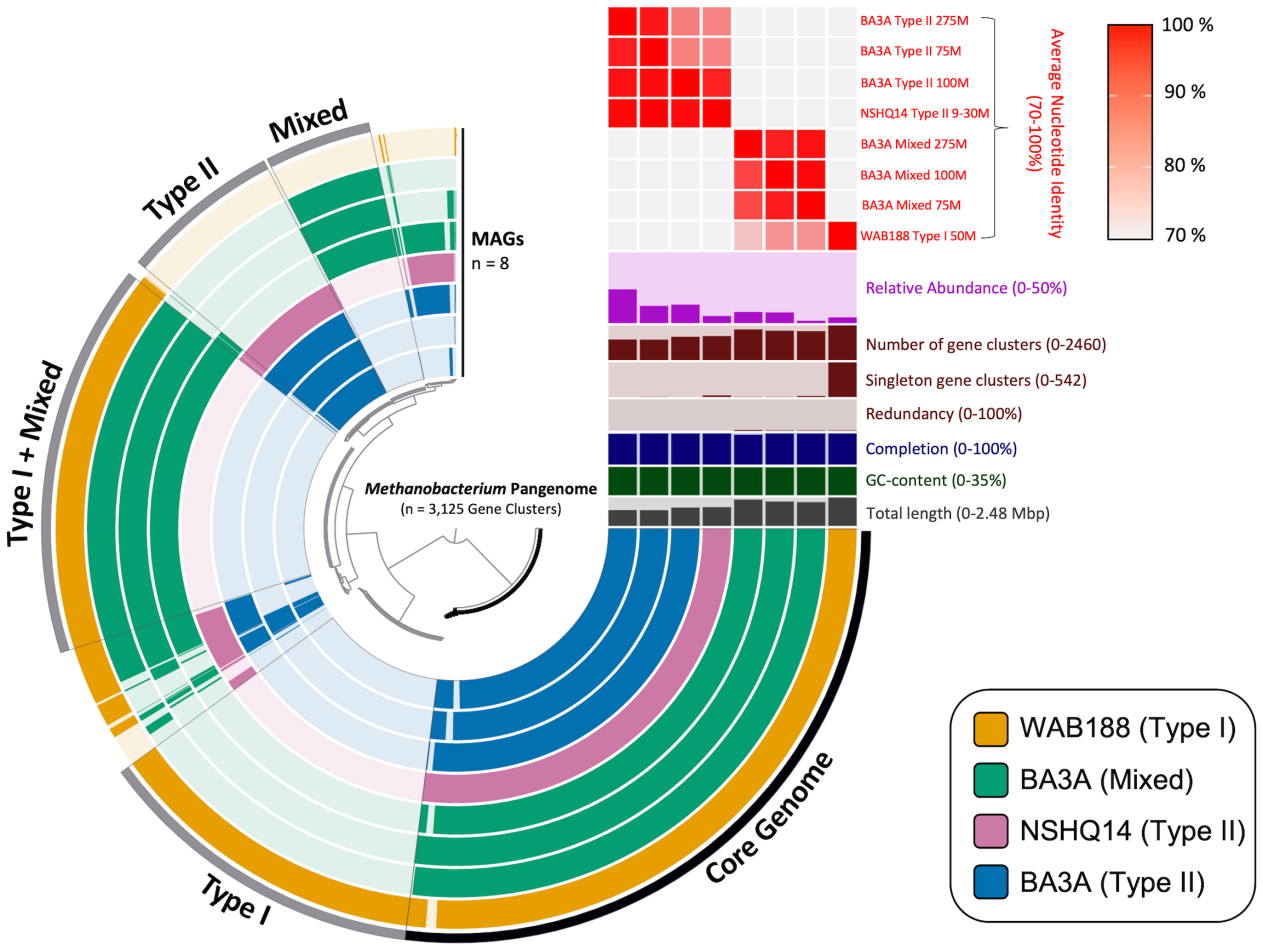
Visual depiction of the pangenome of *Methanobacterium*-affiliated metagenome assembled genomes (MAGs) recovered from subsurface fracture fluids from the Samail Ophiolite, Oman. The inner radial dendrogram shows the 3,125 protein-encoding genes homologs in the pangenome, clustered by presence/absence across MAGs. The 8 MAGs of the three different population “types” are plotted on the innermost 8 layers (270° arcs), ordered and spaced to reflect groupings based on genomic composition. The presence of gene clusters are indicated by filled and colored bars; unshaded bars indicate the lack of a gene cluster in a MAG. Gene clusters belonging to the core genome shared across all MAGs and environmental accessory genomes are binned and labeled with the according specificity on the outside of the outermost layer. The top right portion above the pangenome contains relevant information for each MAG, with the y-axis limits contained within the parentheses. The relative abundance is representative of each *Methanobacterium* MAG’s percentage of overall reads within its corresponding metagenome.

Pangenomic analysis revealed sets of genes intrinsic to all *Methanobacterium* populations that may be vital in the subsurface serpentinized environment. The entire metapangenome of *Methanobacterium* MAGs contains 3,125 gene clusters, of which 36% (1,122 gene clusters) occupy the core genome (Figure 2). The accessory genome of each population appears to decrease in size when the population is found in more hyperalkaline conditions – 17% of total gene clusters (542 gene clusters) in the accessory genome of Type I, 6% (173 gene clusters) in the accessory genome of the Mixed population, and 8% (260 gene clusters) in the accessory genome of the Type II population. To further understand the relationship between the three populations, the average nucleotide identity (ANI) was calculated to determine the genome similarity between each MAG, and is represented in the upper right heatmap of the metapangenome (Figure 2). Results further highlight the distinction of three *Methanobacterium* populations. Of particular note, the Mixed population only shares a stronger genome similarity with the Type I MAG (76 to 82% ANI) than compared to the Type II MAGs (28 to 41% ANI). This finding corroborates the likely short evolutionary separation of this population into hyperalkaline conditions.

### Core genome

The core genome of the eight *Methanobacterium* MAGs (the bottom right portion of the metapangenome) represents all of the genes present among all observed populations (Figure 2). Metabolic reconstruction of annotated genes includes the necessary proteins to carry out the essential steps of the methanogenesis pathway, converting CO_2_ into CH_4_. Additional genes and gene clusters of interest can be inspected in Supplementary Table 1. All *Methanobacterium* MAGs contained the genes for trehalose-6-phophatase synthase and ureidoglycolate dehydrogenase, which serve as possible mechanisms to overcome energy limiting conditions (Kyryakov et al., 2012; Bird et al., 2019).

Comparison of all *Methanobactierum* MAGs to publicly available *Methanobacterium* genomes revealed that only six genes were functionally enriched specifically to the MAGs collected from the Samail Ophiolite with an adjusted q-value < 0.05 (Supplementary Table 2). Some of these genes included CRISPR/Cas associated proteins, mannose-6-phosphate isomerase, and zinc-dependent alcohol dehydrogenase. An additional nine gene annotations were enriched for the Oman MAGs and found in only 10% or less of external genomes. Of note, these annotations included a DNA protection under starvation (DPS) family protein, the zinc-exporting ATPase (*zntA*), and an ABC-type iron exporter (*fetB*; Supplementary Table 2).

### Accessory genomes and functional enrichment

**Figure 3:**
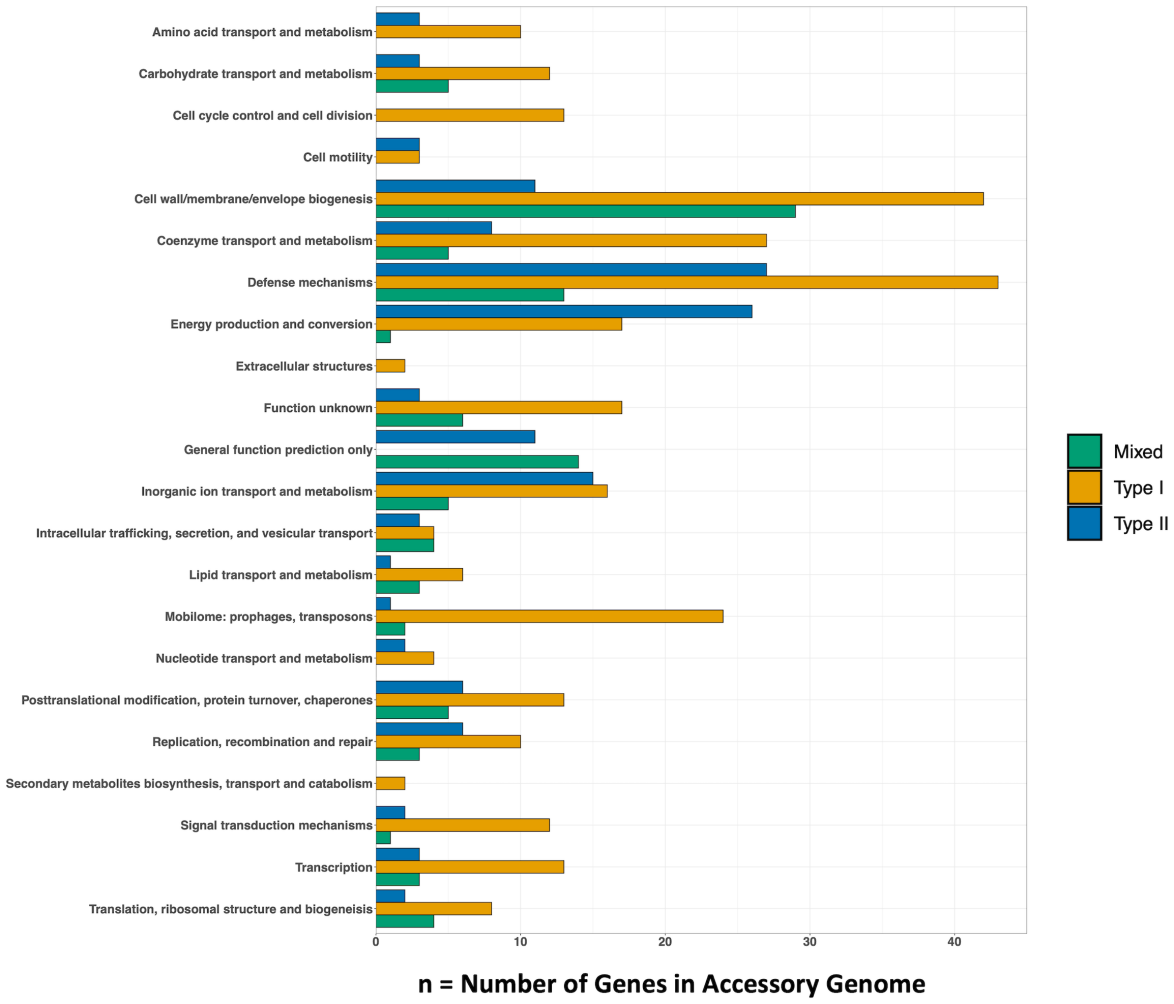
The number of genes within each *Methanobacterium* population’s accessory metagenome assembled genome. The COG20 Category is listed on the y-axis and the x-axis denotes the number of genes that make up the category. The full list of genes found in the top categories of each population can be found in Supplementary Table 4.

**Figure 4:**
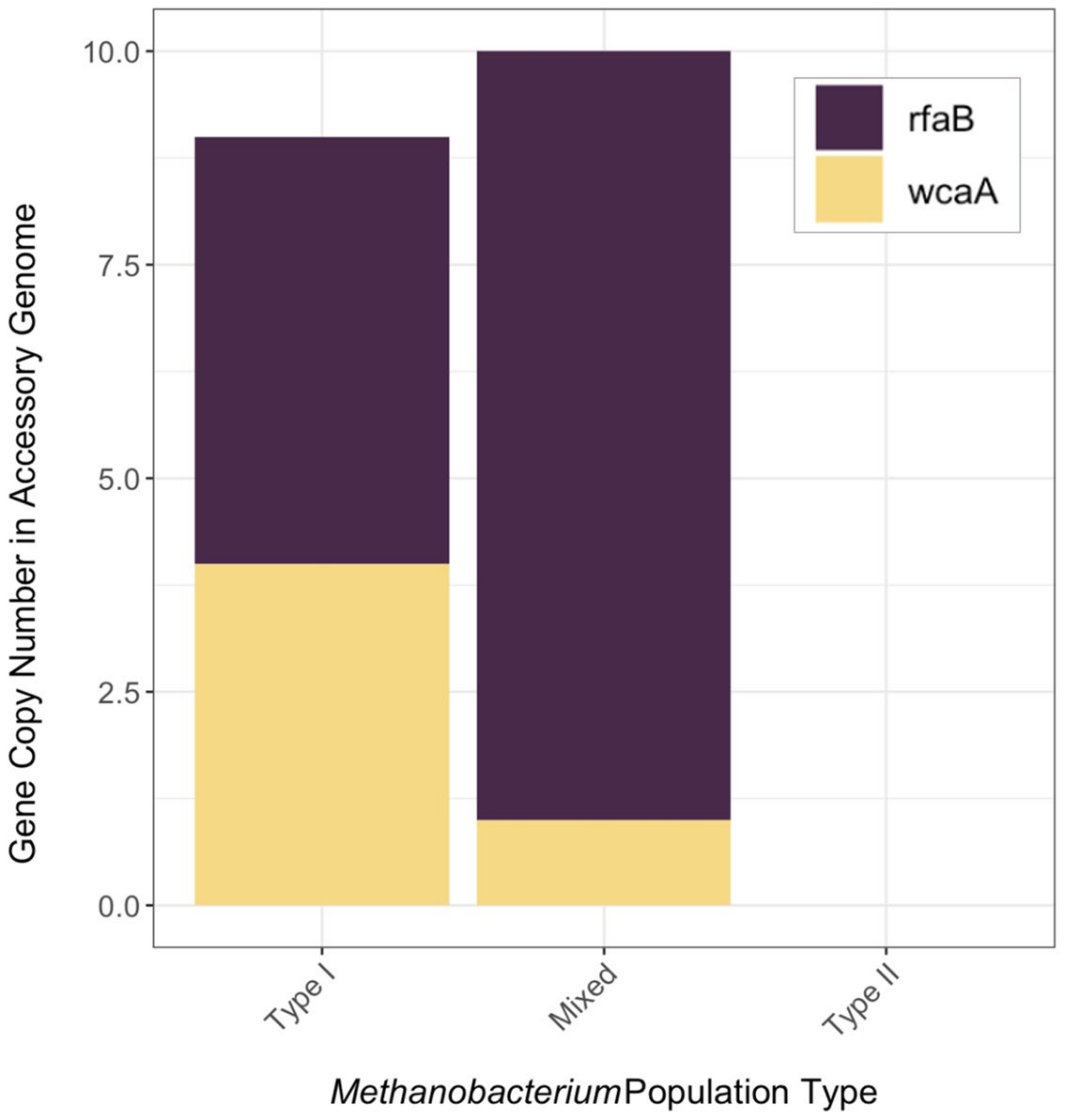
The gene copy number of genes (*rfaB* and *wcaA*) encoding glycosyltransferase in the accessory metagenome assembled genomes of each *Methanobacterium* population. The full list of genes within the accessory genome of each population can be found in Supplementary Table 4.

Genes that were present only in the accessory genome of each *Methanobacterium* population were extracted to identify adaptations to their environmental conditions. The number of genes present in each accessory genome were categorized into 22 observed Clusters of Orthologous Genes (COG) categories (Figure 3). The distribution of genes within accessory genomes discussed below reflects the gene total that falls within one of the COG categories. Additional genes that did not receive functional annotations were not included in the summary of genes belonging to these categories from each *Methanobacterium* population, as this does not reflect the total gene count within the accessory genomes found from each population. The Type I population displayed the largest number of accessory genes annotated by the COG database (*n* = 133) with most genes being placed within the general function prediction only, defense mechanisms, and cell wall/membrane/envelope biogenesis categories. The Type I population contains a large number of genes (*n* = 19) belonging to the radical SAM superfamily within the general function prediction category. Within the cell wall category, the Type I population contained 9 copy numbers of glycosyltransferase, of which 5 were encoded as glycosyltransferases involved in cell wall biosynthesis UDP-D-galactose:(glucosyl) LPS alpha-1,6-D-galactosyltransferase *rfaB* and 4 were annotated as putative colonic acid biosynthesis glycosyltransferase *wcaA* (Figure 4). Many genes appeared to be functionally enriched in the Type I MAG, however, this could be due to the larger genome size leading to an equally as sizeable accessory genome. The functional core of this genome did not reveal any clear distinction between the annotated genes and the environmental conditions observed at WAB188 (Supplementary Table 3).

Comparatively, the Mixed population’s accessory genes with COG functional annotations decreased in size (*n* = 107). The cell wall/membrane/envelope biogenesis COG category was the Mixed population’s standout accessory group with a total of 29 genes. Similar to Type I, the Mixed population contained 10 gene copy numbers of glycosyltransferase, making up more than a third of its dominant COG category. However, the Mixed population encoded only 1 copy of the *wcaA* gene while the rfaB stood out with the remaining *9* copy numbers (Figure 4). Additionally, this dominant category contained the spore coat biosynthesis proteins *spsFG*. The functional enrichment analysis of the Mixed population revealed 20 genes that were not present in any of the other two *Methanobacterium* populations (Supplementary Table 3). These gene annotations appeared to be related to DNA repair or stress and included DNA glycosylase *alkD*, recombinational DNA repair protein *RecF*, and the enamine deaminase of the YjgF/YER057c/UK114 family *ridA*. Additional genes for the presence of lipoprotein and cell wall biosynthesis included teichoic acid biosynthesis protein *tagB*, lysophospholipase *pldB*, and the spore coat biosynthesis proteins *spsFG*. Two acetyltransferases were identified as n-acetyltrasnferases *yhbS* and *rimL*, as well as the ADP-ribosylglycohydrolase *draG*.

The Type II population contained accessory genes (*n* = 130) with the greatest number of genes belonging to the energy production and conversion and defense mechanisms COG categories. Distinct from the other *Methanobacterium*, the Type II population contained genes encoding for type I (*n* = 9) and III (*n* = 1) restriction enzymes. Inspection into the cell wall/membrane/envelope category revealed no annotations for glycosyltransferase in the Type II population. The functionally enriched gene annotations revealed 23 genes that were unique to the Type II population (Supplementary Table 3). Distinct transporters included the ABC-type Fe^3+^ transport system *afuA* and an oxalate/formate antiporter *oxlT*. Potential antimicrobial and detoxification genes included the membrane protein *ydbS*, Zn-dependent glyoxylase *phnB*, cephalosporin hydroxylase *cmcI*, and bacterial immunity and signal transduction membrane protein *SdpI*. Additional genes that were functionally enriched within the Type II MAGs were aconitase hydratase (*acnA*) and isocitrate dehydrogenase (*Icd*) proteins which are responsible for carrying out steps in the tricarboxylic acid (TCA) cycle, though no other gene annotations were detected to form a complete TCA cycle.

### Transporters

**Figure 5:**
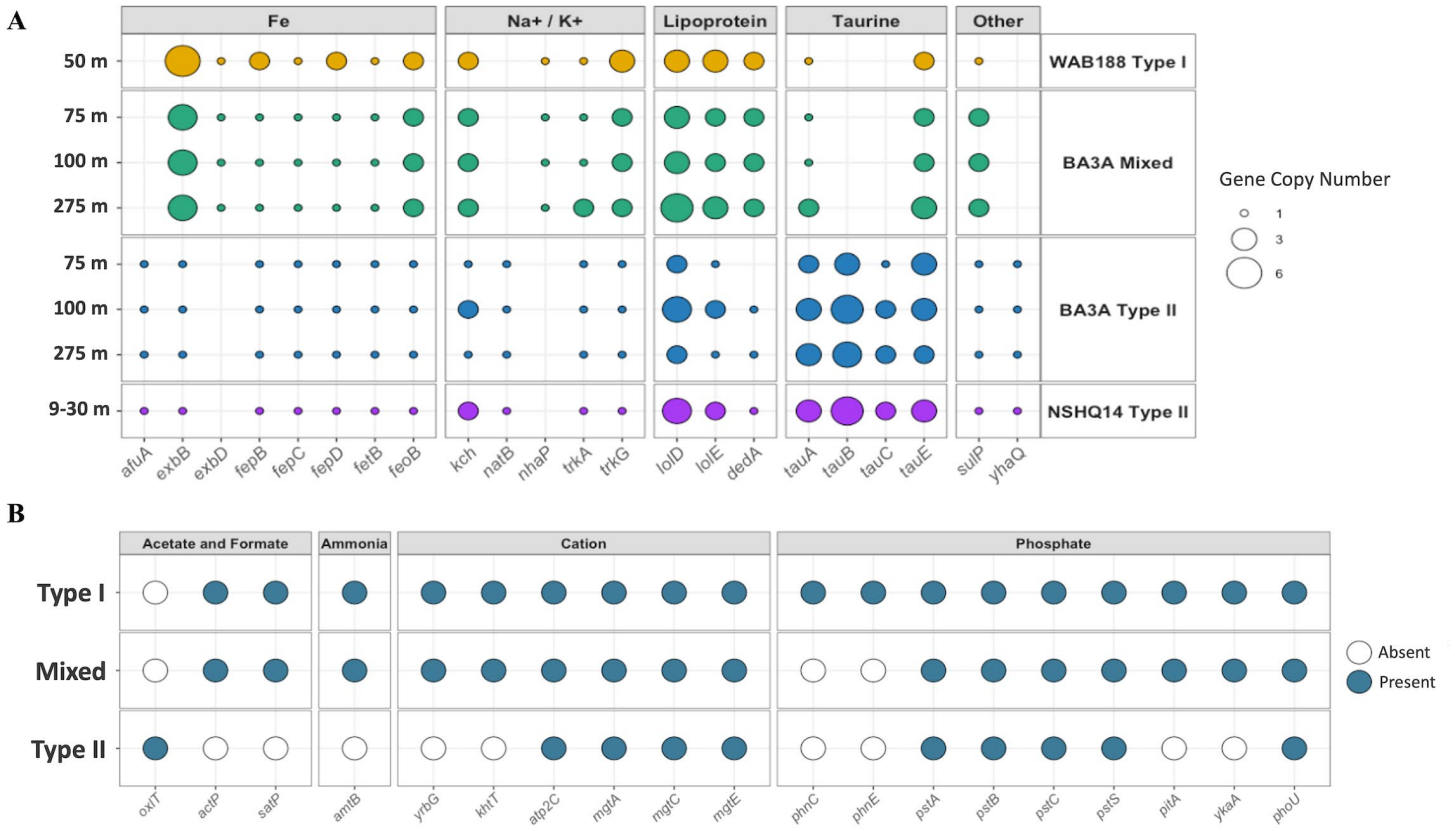
**(A)** The gene copy number of genes involved in transport mechanisms for various metabolites. The x-axis denotes the gene and are faceted (above) into custom transporter categories. The y-axis represents each MAG at the depth in which it was collected and is faceted (on the right) from the borehole in which it was sampled and the *Methanobacterium* population it belongs to. Each dot is also sized by the gene copy number. **(B)** The presence or absence of additional transporter genes across each population type. The presence of a gene is indicated by a filled in dot and represents all MAGs within the population containing the presence of the gene. The x-axis represents the transporter genes and are faceted (above) into custom categories. The y-axis represents the *Methanobacterium* population.

Gene annotations for different transports were inspected for their presence or absence across the different *Methanobacterium* populations in order to resolve potential adaptations through pH homeostasis or circumventing low nutrient availability. Transporter encoding genes were placed into custom categories defined by their gene function and included: Fe, Na^+^/K^+^, Lipoprotein, Taurine, Other, Acetate and Formate, NH_4_^+^, Cation, and Phosphate transporters (Figure 5). All *Methanobacterium* populations shared the presence of all but two Fe transporters. Only the Type II population possessed the *afuA* Fe^3+^ transporter while exhibiting no detection of the *exbD* subunit. The Mixed and Type I population contain both *exb* subunits that are part of an iron uptake complex to transport ferrous iron in the form of siderophores (Noinaj et al., 2010). All *Methanobacterium* populations also contain the *fepBCD* iron siderophore complex (Schalk and Guillon, 2013). Similarly for Na^+^/K^+^ transporters, the Type II MAGs contained the presence of all the identified annotated genes for these transporters, but demonstrates the only presence of the *natB* component of the sodium transport system. Interestingly, the *natA* subunit component was not detected in any of the Type II MAGs. The Type I and Mixed populations contain the only detection of the *nhaP* sodium-hydrogen antiporter, which was absent in the Type II population.

The gene copy number of lipoprotein transport gene annotations was much larger in the Type I and Mixed population compared to the Type II population. Specifically, the *lolE* lipoprotein transport subunit and the *DedA* family protein involved in a variety of cell membrane transport and functions are 2x in gene copy number abundance of the Type I and Mixed populations compared to the Type II populations. Conversely, only the Type II population demonstrates the presence of all three *tauABC* subunits in ≥2x gene copy number. However, all populations share the presence of the *tauE* sulfite exporter. Within the “Other” category of transporters, only the Type II population contained the putative ABC-transporter *yhaQ*. All *Methanobacterium* MAGs shared the presence of a sulfate permease (*SulP*) gene. The *SulP* gene from the Type II population was resolved through BlastP analysis and identified as a putative sulfate transporter. Further BlastP analysis did not resolve the *SulP* transporter from the Type I population, and was broadly characterized as an inorganic anion transporter belonging to *Methanobacterium* with 76% identity. However, only the Mixed population possessed both of these *SulP* encoded proteins.

The presence or absence of genes was extended to additional categories to further highlight potential adaptions in hyperalkaline fluids by the *Methanobacterium* populations (Figure 5). The Type II population contains the only detected formate transporter annotated as an oxalate:formate antiporter. Additionally, it appears that the Type II population does not possess an acetate transporter, while the Type I and Mixed populations share the same transporters, *actP* and *satP*, for potential acetate assimilation. Similarly, the Type II population demonstrates the absence of the ammonium transporter *amtB* and the potassium/hydrogen antiporter *khtT*. Only the Type I *Methanobacterium* population displays the presence of the *phnCE* subunits of the phosphonate transporters. The Type I and Mixed population share gene annotations for phosphate and metal symporter *pitA* and phosphate transport regulator *ykaA*. Additional gene absence, presence, and copy number are illustrated in Figure 5.

### Carbon cycling

**Figure 6:**
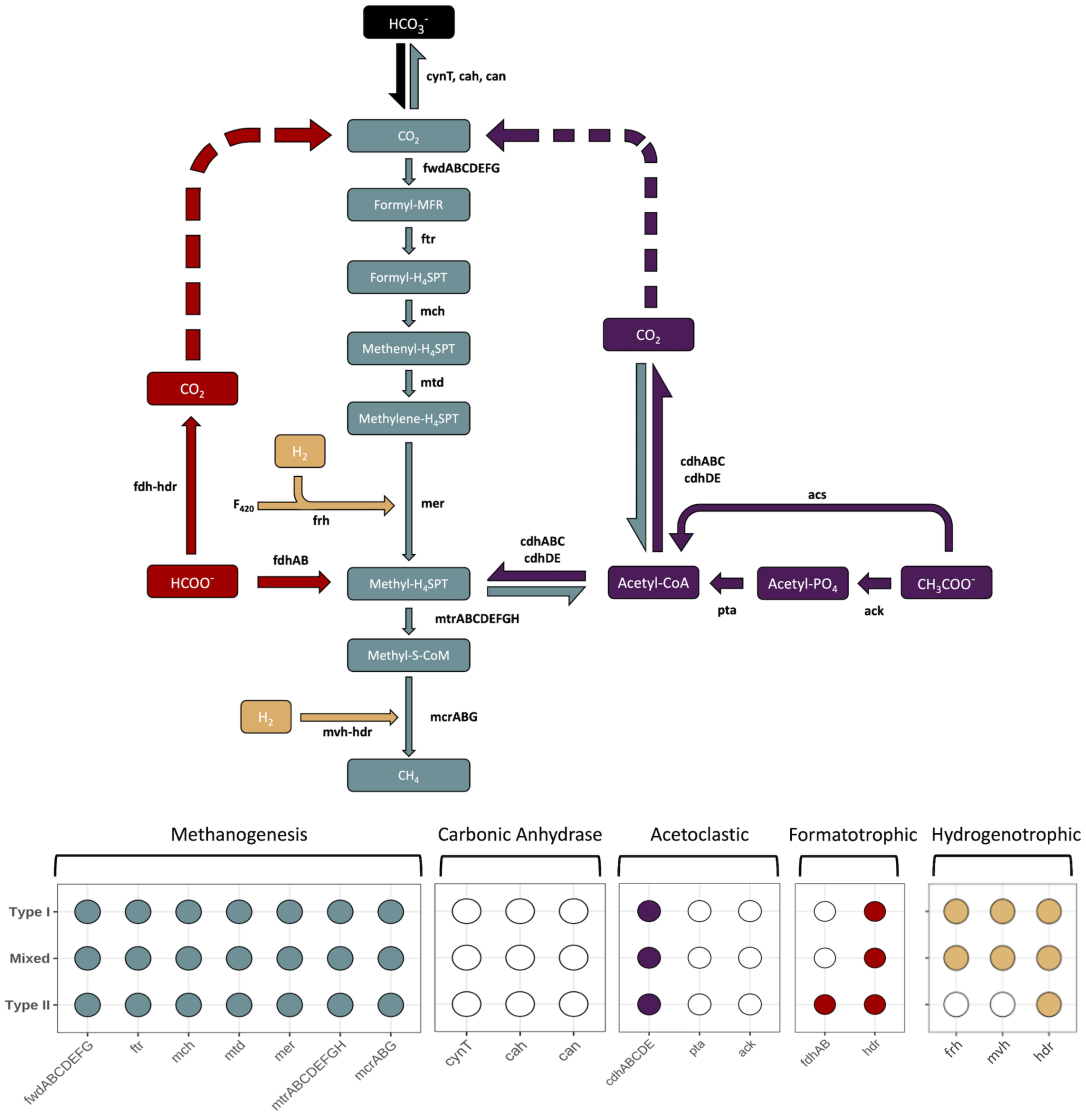
The different enzymatic pathways for the core methanogenesis pathway (gray), hydrogenetrophic (tan), acetoclastic (purple), formatotrophic (red) or steps involving conversion of bicarbonate into CO_2_ for methanogenesis (black). Carbon substrates, intermediates, and products appear in rectangular boxes, while enzymes are in black lettering next to arrows that point in the direction of their end product. Dashed arrows indicate the potential use for the pathway to allow for intracellular CO_2_ to be generated and subsequently reduced to CH_4_. The enzymatic component of heterodisulfide reductase (hdr) is included in both formatotrophic and hydrogenetrophic pathways because each due to its similar function, but does not represent a different gene. The bottom of the figure contains a presence/absence diagram, where circles are filled in to represent the presence of a gene in the respective pathway and a white, unfilled circle indicates the absence of a gene. The rows of the presence/absence diagram are representative of each *Methanobacterium* population. Figure inspired by Carr et al. (2018). Genes: fwd, formylmethanofuran dehydrogenase; ftr, formylmethanofuran:H4SPT formyltransferase; mch, methenyl-H4SPT cyclohydrolase; mtd, F420-dependent methylene H4SPT dehydrogenase; mer, F420-dependent methylene-H4SPT reductase; mtr, methyl-H4SPT:coenzyme M methyltransferase; mcr, methyl-coenzyme M reductase; ack, acetate kinase; pta, phosphate acetyltransferase; acs, acetyl-CoA synthetase; cdh, carbon monoxide dehydrogenase; fdh, formate dehydrogenase; hdr, heterodisulfide reductase; mvh, methyl viologen-reducing [NiFe]-hydrogenase; frh, coenzyme F_420_ [NiFe]-hydrogenase; F_420_, coenzyme F_420_. Carbon compounds: CO_2_, carbon dioxide; formyl-MFR, formyl-methanofuran; formyl-H4SPT, formyl-tetrahydrosarcinapterin; methenyl-H4SPT, methenyl-tetrahydrosarcinapterin; methylene-H4SPT, methylene-tetrahydrosarcinapterin; methyl-H4SPT, methylene-tetrahydrosarcinapterin; methylene-S-CoM, 2-(Methylthio)ethanesulfonate; Acetyl-CoA, acetyl-Coenzyme A; Acetyl-PO_4_, acetylphosphate; CH_3_COO^−^, acetate; HCOO^−^, formate.

Multiple pathways for carrying out methanogenesis were investigated, and include suggested mechanisms from previous studies conducted at the Samail Ophiolite (Fones et al., 2021; Kraus, 2021). All *Methanobacterium* MAGs were queried for proteins involved in hydrogenotrophic, acetoclastic, and formatotrophic methanogenesis along with carbonic anhydrase to potentially convert bicarbonate into bioavailable CO_2_. The variety of pathways for methanogenesis were examined in order to determine adaptability to available carbon substrates (Figure 6). Type I and Mixed *Methanobacterium* MAGs contained the full suite of proteins encoded to carry out the hydrogenotrophic methanogenesis pathway, reducing CO_2_ in the presence of H_2_ to CH_4_ (Figure 6). In line with previously reported work, only Type II *Methanobacterium* populations encoded the presence of formate dehydrogenase (*fdhAB*), which allows for potentially using formate as an alternative and sole carbon source to circumvent low DIC in hyperalkaline conditions (Fones et al., 2021). None of the proteins required for carbonic anhydrase proteins were detected in any of the *Methanobacterium* populations. Furthermore, none of the MAGs encoded for the *ack*/*pta* proteins that are typically found in acetoclastic methanogens (Stams et al., 2019). All *Methanobacterium* MAGs contained the acetyl-CoA synthetase (*acs*) protein which allows for the conversion of acetate into acetyl-CoA (Hattori, 2008). However, the *Methanobacterium* populations are likely assimilating carbon from acetate for biosynthesis of various cell components rather than being incorporated into CH_4_ production (Oberlies et al., 1980).

## Discussion

### *Methanobacterium* core genome and shared strategies

*Methanobacterium* populations play a significant role as the primary methane producer in the hyperalkaline subsurface fluids of the Samail Ophiolite (Fones et al., 2019; Kraus, 2021). While previous work has elucidated a potential adaptation for circumventing DIC limitation in the subsurface by use of formate by *Methanobacterium*, more work is needed to fully unravel the strategies *Methanobacterium* uses to adapt to both a high pH and a nutrient limited environment (Fones et al., 2021). In this study, eight MAGs across various geochemical gradients have been exploited to investigate metabolic strategies for living in the conditions experienced in the most hyperalkaline wells. Results from phylogenomic analysis and ANI metrics reveal three distinct *Methanobacterium* populations exist in the subsurface serpentinizing fluids of the Samail Ophiolite (Figures 1, 2). Previous work in the Samail Ophiolite suggests that the diversification of methanogens and acetogens can be reflected by the fluid type in which those populations exist (Fones et al., 2021; Colman et al., 2022). This work corroborates the distinction between Type II *Methanobacterium* populations that exist in the hyperalkaline fluids and Type I populations collected from neutral waters. However, the most intriguing discovery includes the identification of a third Mixed *Methanobacterium* population. The Mixed *Methanobacterium* population likely represents an intermediary in the evolutionary history from Type I to Type II lineages. Interestingly, the Mixed population appears only within the most hyperalkaline borehole and was not detected in any other fluid source. This raises key questions regarding why a *Methanobacterium* population that is likely to be better suited to exist in more hospitable conditions only subsists within the most unfavorable geochemical regimes within the serpentinizing subsurface.

Evidence for genome streamlining is indicated by the decrease in genome size between methanogenic populations from neutral and hyperalkaline conditions. When organisms face selective pressures from their environment, a reduction in genome size allows for lessening the costs of energetics associated with the replication of their genome (Giovannoni et al., 2014). The DIC limited and high pH conditions found in the more serpentinizing impacted fluids results in both nutrient limitation and osmotic stress of organisms, and would suggest genome streamlining acts as a favorable adaptation to hyperalkaline fluids. The Type I *Methanobacterium* MAG displays the largest genome size and likely receives less environmental stress, given that surficial CO_2_ can more readily mix into the fluids and prevent carbon limitation. The Type II population contains the smallest genome sizes, and reflects previous work where these hyperalkaline-adjusted-organisms have reduced their genome size to cut down energetic costs (Fones et al., 2021). The genome sizes of the Mixed *Methanobacterium* population are greater than Type II populations, yet smaller than Type I. This genome size variance could suggest that the Mixed population have not fully adapted to the nutrient limited and hyperalkaline conditions, and therefore have not yet fully streamlined to minimize energy demands. The presence and absence of the various transporter gene annotations between all *Methanobacterium* MAGs may highlight how the Type II population keeps necessary transporters for the metabolites that are under greatest demand in the hyperalkaline fluids (Figure 5). Additionally, genome streamlining may be the dominant strategy for adapting to hyperalkaline conditions observed by the *Methanobacterium* populations of the Samail Ophiolite compared to external *Methanobacterium* genomes. This potential adaptation strategy becomes emphasized by the lack of genes determined from functional enrichment analysis belonging specifically to the *Methanobacterium* populations from Oman. A reduction in genome size will decrease the number of shared genes across all three populations, and therefore makes it difficult to distinguish unique genes or adaptation strategies for circumventing the subsurface serpentinized fluids from the Samail Ophiolite. Instead, greater focus on the differences between the three populations identified within this study provide greater resolution of potential lifestyle strategies invoked by changes in local geochemistry across different boreholes and depths.

The pangenomic comparisons among these three *Methanobacterium* species allows for investigation of shared survival strategies and how each has adapted unique capabilities to overcome limiting conditions in the serpentinizing subsurface. Pangenomic analysis revealed sets of genes intrinsic to all *Methanobacterium* populations that may be necessary in the subsurface serpentinized environment. Trehalose-6-phophatase synthase was found in all *Methanobacterium* MAGs (and only 66% of publicly available genomes not from Oman), and has been suggested as a low-energy state strategy in other subsurface systems (Argüelles, 2000; Bird et al., 2019). This protein has been reported from other energy limiting systems in sediments of the Baltic Sea and Antarctic soils, where metagenomic sequencing has detected trehalose synthase to aid in stabilizing cellular membranes against osmotic stresses (Koo et al., 2018; Bird et al., 2019). This protein has been suggested to help by producing trehalose which prevents accumulation of degraded proteins, slow replication rates, and increase cellular longevity (Brauer et al., 2006; Kyryakov et al., 2012; Sipes et al., 2021).

Additionally, all *Methanobacterium* MAGs contain ureidoglycolate dehydrogenase which is involved in the degradation of allantoin in order to access detrital DNA (Bird et al., 2019; Sipes et al., 2021). Though it has been previously reported that ureidoglycolate dehydrogenase is employed under nitrogen starvation, work quantifying the cycling of nitrogen within the subsurface fluids of the Samail Ophiolite is replete (Rempfert et al., 2023). However, the dominant nitrogen species in the hyperalkaline fluids is NH_4_^+^. Additionally, the Type II population does not contain the presence of an NH_4_^+^ transporter (*amtB*) that the Mixed population possesses (Figure 5). The advantage for the Type II *Methanobacterium* to not contain a NH_4_^+^ transporter is not clear. In order to maintain internal cytoplasmic pH balance, the Type II population may have adapted to not assimilate NH_4_^+^ due to the cationic charge. NH_4_^+^ has also been demonstrated to become toxic to methanogens in bioreactors in large concentrations, yet not at the levels reported here or at the pH conditions seen at the Samail Ophiolite in hyperalkaline fluids (Capson-Tojo et al., 2020). Further, in pH 11 fluids the dominant nitrogen species will likely be NH_3_, the uncharged form. *Methanobacterium* may be able to acquire NH_3_ that passes through the cell without the need of an ion gradient. While the lack of an NH_4_^+^ transporter in the Type II population in not apparent, ureidoglycolate dehydrogenase may prove an effective strategy to overcome potential nitrogen limitation. The presence of these two encoded proteins indicates that *Methanobacterium* may employ strategies that involve maintaining a low-energy state for survival and scavenging during periods of nutrient starvation (Liang et al., 2019; Sipes et al., 2021). Within the hyperalkaline fluids, DNA scavenging tactics for phosphate may take place since DNA can serve as a source for phosphorous. Additionally, phosphorous species will precipitate out in the hyperalkaline Ca^2+^ and OH^−^ rich fluids further limiting it is availability. Scavenging for DNA supports an avenue for alternative phosphorous uptake in the hyperalkaline fluids that have been suggested to be phosphorous limited (Kraus, 2021; Rempfert et al., In Review). Maintaining low energy states may also explain how both the Type II and Mixed *Methanobacterium* populations comprise the greatest relative abundance within the more extreme fluids. By potentially employing strategies to reduce replication rates and lower energetic costs, the methanogenic populations within the hyperalkaline conditions may be able to retain greater cell concentrations, while other microbial members are unable to meet the energetic demands and cannot sustain replication rates to make up a significant portion of the subsurface community. In comparison, conditions within WAB188 contain inputs of surface derived DIC and greater access to other nutrients. Though, it is possible that Type I *Methanobacterium* are being out-competed by other microbial members for resources and may need to employ these survival techniques against contending microorganisms. These findings suggest that all *Methanobacterium* populations might use DNA scavenging and low-energy state strategies in order to overcome nutrient limitations imposed by environmental pressures or community competition and thus adapt to subsurface serpentinizing conditions.

Functional enrichment analysis revealed that all *Methanobacterium* populations share the iron exporter *fetB* that is only found in 3.5% of other observed *Methanobacterium* genomes (Supplementary Table 2). This protein has been reported to play a role in providing resistance to oxidative stress through iron homeostasis (Nicolaou et al., 2013). Reactive oxygen species such as the hydroxyl radical (•OH) can be threatening to DNA and lipids (van Erk et al., 2023). These reactive oxygen species can be formed through redox reactions or from the Fenton reaction in the presence of iron (Burns et al., 2010). Given the greater concentrations of ferrous iron from serpentine minerals, dissolved iron, and the dominance of OH^−^ primarily in the hyperalkaline fluids, the role of iron homeostasis may play a role in providing defense against oxidative stress imposed on the *Methanobacterium* populations not commonly observed from the external *Methanobacterium* genomes originating from non-serpentinizing environments. However, the generation of reactive oxygen species in the presence of iron usually results from the abundance of oxygen, which is not replete within the hyperalkaline subsurface (Burns et al., 2010). Therefore, it is not clear still why *fetB* would provide an advantage unless another oxidative stress from other geochemical analytes is imposed on the *Methanobacterium* MAGs observed in this study. Though reactive oxygen species may play a role in the hyperalkaline subsurface, the role of iron transport from *Methanobacterium* populations is influenced by the necessity for [NiFe]-hydrogenases that are requisite for methanogenesis. All *Methanobacterium* populations contain the *exbB* transporter gene, yet the Mixed and Type I population contain an incredibly large gene copy number compared to the Type II MAGs (Figure 5). This *exbB* protein is involved in the uptake of iron through siderophore uptake (Schalk et al., 2004). The Type II population contains the only presence of the putative iron ABC transporter *afuA*, which may allow this population an advantage toward accruing iron in hyperalkaline conditions. Overall, greater efforts are needed to resolve the microbially mediated iron acquisition and trafficking in serpentinizing systems.

### Unique adaptation strategies resolved from accessory genomes and functional enrichment

The three different *Methanobacterium* populations’ accessory genomes were evaluated to investigate unique gene clusters and individual genes that may promote an advantage to overcome environmental challenges in the subsurface of the Samail Ophiolite. Initial trends can be delineated from the three methanogenic populations between the dominant COG categories in which the most accessory genes fall under. Functional enrichment analysis further provided insight into the functional core set of genes unique to each population to reflect adaptations imposed by either hyperalkaline or circumneutral fluid conditions.

Type II *Methanobacterium*’s accessory genome demonstrates preference toward “Energy production and conversion” and “Defense Mechanisms.” It is likely that the dominance of genes related to energy production is in line with Type II *Methanobacterium* being capable of potential formatotrophic methanogenesis due to the formate dehydrogenase genes (*fdhAB*) only appearing in the accessory genome of the Type II population. This may suggest that Type II *Methanobacterium* are well adapted to hyperalkaline fluids, and may be able to take advantage of unique sources of carbon and other nutrients to circumvent energy limitations. The presence of genes related to defense mechanisms is intriguing since the threat of potential viral infections and microbial competition may not be as extensive in hyperalkaline fluids, yet this needs greater efforts to resolve why this may aid Type II *Methanobacterium*. This defense mechanisms category is dominated by genes encoding for restriction enzymes and components of toxin/antitoxin systems which dually play a role in viral protection and potentially against other microorganisms (Supplementary Table 4). The 10 gene copy numbers of restriction enzymes belonging to the Type II population may play a further role in DNA scavenging strategies. An increase in restriction enzyme levels has been proposed as a mechanism mainly to protect cells from viral infection (Loenen et al., 2014). However, phosphate is a limiting nutrient in the hyperalkaline subsurface, often below a 5 μM detection limit (Rempfert et al., 2017, In Review. Table 1). Thus, restriction enzymes could function as a mechanism for cleaving detrital DNA as a source for phosphate uptake.

Intriguingly, the significant quantity of encoded proteins related to antimicrobial resistance and detoxification from the Type II population suggests a unique survival adaptation. The *ydbS* protein is reported to be involved in general resistance to antimicrobials, and *cmcI* is linked to detoxifying cephalosporins which act as compounds to disrupt cell wall synthesis (Öster et al., 2006). The *SdpI* protein is known to protect against a toxin that lyses cells open for feeding off the nutrients released from these dead cells. In a strain of *Bacillus subtulis*, this organism has been shown to produce a cannibalism toxin under nutrient limitation that will kill and lyse open cells within its own population (Povolotsky et al., 2010). The cells that display the activated *SdpI* protein are able to recognize the toxin and are immune to being lysed, and gain the opportunity to uptake nutrients from localized dead cells or scavenged DNA (Ellermeier et al., 2006). While the ability for Type II *Methanobacterium* to produce this toxin was not confirmed, this population may have acquired the resistance to a toxin possibly released by another subsurface microbial member. Therefore, we conjecture that the Type II *Methanobacterium* may be employing an adaptive strategy by scavenging from dead cells as a mechanism for potentially overcoming nutrient limitation, a viable approach due to phosphate limitation in the hyperalkaline fluids.

The Type I population contains the greatest number of unique genes in the “General function prediction only” category, though also shows great abundance in the “Cell wall/membrane/envelope biogenesis” and “Defense mechanisms” COG categories. Speculating from these categories, Type I *Methanobacterium* may require greater efforts for defending against viral infections or competition from other microbial members. Subsurface fluids with neutral pH conditions have been reported to contain a higher diversity and richness of microorganisms (Brazelton et al., 2012; Rempfert et al., 2017). These fluids typically are composed of a larger presence of stronger oxidants and limited by reductants such as H_2_. Even more, at contact wells such as WAB188 where our Type I MAG exists, fluid mixing allows for greater fluctuation of geochemistry. Type I *Methanobacterium* populations might need to be flexible to the non-stagnant fluid chemistry observed at contact wells. Therefore, the greater competition of other microbial members for available reductants may require Type I *Methanobacterium* to employ ways to circumvent thermodynamic competition. While the viral community has not yet been explored in the subsurface fluids of the Samail Ophiolite, it is likely that the abundance of viral members would reflect similar trends in the microbial distribution among serpentinizing fluids. Therefore, defensive strategies to hold off viral infection may be greater in the Type I fluids compared to the deep hyperalkaline environments.

Interestingly, despite both Type II and Mixed *Methanobacterium* occupying hyperalkaline fluids, only the Mixed population’s accessory genome is predominantly comprised of genes that fall under the “Cell wall/membrane/envelope biogenesis” COG20 category. The presence of spore coat polysaccharide biosynthesis proteins (*spsFG*) have been reported for entering a state of stasis until conditions become more favorable may imply sporulation as a mechanism for protecting Mixed populations (Liang et al., 2019). Sporulation has not been observed as a strategy imposed by methanogens, yet these genes may suggest a beneficial role to the membrane structure of the Mixed population to serve as an environmental stress defense. As the Mixed *Methanobacterium* population represents an intermediary between Type I and Type II methanogens, it is possible that the Mixed population have adapted to hyperalkaline conditions requiring greater cell membrane integrity preventing cell disruption. The functionally enriched genes of the Mixed population may reflect their adaptation mechanisms necessary for subsisting in the highly reduced fluids. The Mixed population contains the presence of the *pldB* lipophospholipase which may further help to provide membrane stability. Lipophospholipases, such as those encoded by *pldB*, are responsible for metabolizing lipophsopholipids which are responsible for cell signaling processes and regulation of the cellular membrane structure (Kobayashi et al., 1985). Additionally, it has been shown that lipophospholipases play a role in maintaining lipid homeostasis (Wepy et al., 2019). While these biomolecules have not been studied for their role in alkaline environments, we posit that the Mixed *Methanobacterium* population requires greater effort to organize its cell membrane in order provide membrane stability or support potential collaboration with other microbial members. Cell signaling processes may indicate the Mixed population is participating in biofilm interactions where lipophospholipases regulate intermediary exchanges of metabolites.

Accessory genome results from the Mixed *Methanobacterium* population indicate a role for glycosyltransferases to support niche adaptation within the high pH conditions and coexistence with Type II *Methanobacterium*. The incredible shift from Type I populations containing a near even distribution of the *rfaB* and *wcaA* glycosyltransferases to Mixed populations containing almost entirely *rfaB* signifies the importance of this gene for providing an advantage in the hyperalkaline fluids (Figure 4). Previous work has demonstrated under glucose limitation that the *wcaA* protein requires a greater energetic cost and is not expressed (Pradel et al., 1992; Wang et al., 2020). The lack of any glycosyltransferase genes in the Type II population’s accessory genome further represents the niche differentiation of the Mixed population within the hyperalkaline fluids. The presence of the glycosyltransferase gene *rfaB* may indicate opportunities for biofilm interaction from methanogenic microbial members. The *rfaB* protein has been linked to biofilm formation and is necessary for certain microbes to participate within biofilms (Raaijmakers et al., 2010; Chai et al., 2012; Probst et al., 2014). In addition, the potential interaction with biofilm communities would increase the likelihood for a non-motile mode of habitation for the Mixed population. Postulating upon alternative adaptation strategies, Mixed *Methanobacterium* populations might require cooperation between additional microbial members in order to gain access to DIC. Specifically, other microbial members able to liberate bicarbonate from carbonate mineral veins or generate bioavailable CO_2_ may require the Mixed population to interact directly through syntrophic partnership or interspecies electron transfer. Further transcriptomic analysis and physiological studies are necessary to corroborate the role of other microorganisms possessing carbonic anhydrase able to the speciation bicarbonate or aid methanogens by another metabolic mechanism. The increased gene copy number of *rfaB* in the Mixed *Methanobacterium* population’s accessory genome would suggest biofilm interaction from Mixed *Methanobacterium* MAGs poses as a substantial environmental adaptation to circumvent the lack of available DIC in the hyperalkaline fluids.

The Type II and Mixed populations occupy a large abundance (up to 23.87% and 7.91% relative abundance, respectively) of the overall microbial community within the hyperalkaline fluids within BA3A. We hypothesize that niche differentiation is supporting the ability for coexistence, given the two *Methanobacterium* populations inhabit the same environmental ecosystem with accessory genomes highlighting very different lifestyles. These differences minimize overlap in their functions within the microbial community and allow for successful adaptation within the hyperalkaline fluids. The Type II population appears capable of a metabolic adaptation to utilize formate in the absence of a clear source of DIC. Contrastingly, the enriched number of glycosyltransferases, specifically *rfaB*, within the Mixed population may indicate greater interaction with biofilms or other microbial members. Further, this would suggest Mixed *Methanobacterium* occupy a more sessile mode of existence in order to reduce energetic demands in search for a source of DIC. Alternatively, the Mixed population could attach to abiotic surfaces to potentially acquire a source of nutrients. The lack of any genes related to cell motility within the accessory genome of the Mixed population further supports the possibility of a sessile lifestyle (Figure 3). Ultimately, the augmentation of glycosyltransferases in the Mixed *Methanobacterium* population supports the notion of facilitating strong niche differentiation from the Type II population to enable co-habitation within the hyperalkaline fluids.

### Transporters demonstrate further niche differentiation

The contrast of different gene annotations to related transporters was explored to provide insight into adaptation to hyperalkaline conditions from Type II *Methanobacterium* as well as to compliment how the Mixed population may require additional osmoregulation or nutrient acquisition strategies. The Type II population contained a small subset of transporter genes that were not observed in the other two populations that might provide additional benefit in the hyperalkaline subsurface. The Na^+^ transporter *natB* was only possessed by the Type II population, and may play some role in maintaining osmoregulation. Surprisingly, the Type II population was absent of the *nhaP* Na^+^/H^+^ antiporter and *khtT* K^+^/H^+^ antiporter, which would appear beneficial given the high H_2_ concentration in hyperalkaline fluids. However, this antiporter may not be active at hyperalkaline pH, and may result in gene loss from genome streamlining of the Type II population. Fones et al. (2021) reported on the Mrp-MbH complex (not reported here) that is present in the Type II population and is predicted to help maintain pH homeostasis. Interestingly, the Type II *Methanobacterium* contained the only presence of the full suite of *tauABC* genes responsible for uptake of taurine. Taurine may provide a source of sulfur for Type II methanogens (Kraus, 2021). Sulfate concentrations are an order or two magnitude lesser in the hyperalkaline fluids of NSHQ14 and BA3A compared to WAB188, and taurine may prove as an alternate source of sulfur (Table 1). However, taurine may also serve as an osmoprotectant in order to balance the external osmotic pressure faced in the hyperalkaline fluids (Zhang et al., 2016; Yan et al., 2022). Methanogens and other microbiota have been reported to accumulate soluble organic compounds such as taurine or glycine betain, neutral molecules compatible with metabolic processes and cellular structure. These organic solutes are predicted to provide osmotic balance within the cell against environmental osmotic stress (Farwick et al., 1995; Yan et al., 2022). The ability for Type II *Methanobacterium* to accumulate taurine as a possible osmoprotectant highlights an additional adaptation strategy by this methanogenic population, though the source of taurine within the hyperalkaline subsurface is unclear.

It is additionally perplexing as to why the Type II population only possesses the ATP2C P-type Ca^2+^ transporter shared among all *Methanobacterium* populations and not additional Ca^2+^ transport mechanisms. The Type II population is absent of the yrbG cation:H^+^ antiporter, yet demonstrates the presence of the Mg^2+^ transport system from *mgtACE* genes. One study demonstrated that *M. thermoautotrophicus* was directly dependent on Ca^2+^ availability for methane production and cell growth (Vancek et al., 2006). Ca^2+^ concentrations are incredibly high in the hyperalkaline fluids (3.35–6.2 mM) compared to the neutral fluids (1.21 mM). Additional cation transporters for the Type II *Methanobacterium* population may not be energetically favorable or have pH dependencies that are exceeded in the hyperalkaline conditions and may be excluded due to genome streamlining despite high Ca^2+^ concentrations in hyperalkaline waters. The absence of the NH_4_^+^ transporter in the Type II population is intriguing given the dominant source of nitrogen in the highly reduced fluids is NH_4_^+^. Contrastingly, phosphate may be a limiting nutrient in the serpentinizing subsurface (Kraus, 2021). The Type II population lacks the *ykaA* phosphate transport regulator, which may further indicate that the cells are under constant phosphate limitation and do not require regulation.

The Mixed *Methanobacterium* population demonstrates few transporter mechanisms that would suggest an adaptation or strategy to circumvent the high pH conditions found in borehole BA3A. The greater gene copy number of lipoprotein transporters compliments the presence of functionally enriched lipoprotein gene annotations found in the Mixed population. Specifically, the gene annotations for the *DedA* family proteins are involved in the transport of various phospholipids in order to rearrange the lipid dynamics of the cell membrane and provide improved membrane integrity (Kumar and Doerrler, 2015; Okawa et al., 2021). While the exact function of these proteins is not fully understood, the reoccurring theme of the Mixed population’s focus on cell membrane arrangement suggests this population is actively combating the environmental stress imposed in the hyperalkaline subsurface fluids. The Mixed *Methanobacterium* MAGs also contain an additional sulfate permease gene compared to the other two populations. While the initial sulfate permease protein identified across Type II and Mixed *Methanobacterium* MAGs appears to be involved in sulfate transport, the additional *SulP* protein in the Mixed and Type I population shares homology to a generic inorganic anion transporter from BlastP analysis. Sulfate permeases compose a large family of proteins primarily responsible for sulfate transport, but recently recognized for anion:anion antiport exchange in some homologs (Felce and Saier, 2004). Bioinformatic analysis of various *SulP* genes demonstrated that many of them are fused to carbonic anhydrase homologs, including the bicarbonate transporter belonging to the *SulP* family within a marine cyanobacteria (Felce and Saier, 2004; Price et al., 2004). Further efforts are required to determine if the additional *SulP* protein identified in the Mixed population could act as an anion transporter to circumvent DIC limitation or provide another adaptation strategy in the hyperalkaline, reduced fluids. Physiological studies providing a better understanding of how Mixed *Methanobacterium* acquires cytoplasmic CO_2_ are necessary to inform on how this population contributes to a significant proportion of the relative abundance within the deep subsurface hyperalkaline fluids of borehole BA3A.

### Carbon substrate adaptability

Understanding how methanogens are able to survive in the hyperalkaline conditions despite an obvious source of DIC remains puzzling. Multiple pathways for carrying out methanogenesis were investigated, and include suggested mechanisms from previous studies conducted at the Samail Ophiolite (Kraus, 2021; Fones et al., 2021). Corroboration of Type II *Methanobacterium* MAGs possessing formate dehydrogenase encoded proteins to oxidize formate to generate cytoplasmic CO_2_ matches previous findings and supports an alternative methanogenic pathway to overcome CO_2_ limitations (Fones et al., 2019, 2021). Though, the significant presence of the Mixed *Methanobacterium* population within hyperalkaline waters not containing formate dehydrogenase genes suggests this methanogen population is being supported through another adaptation strategy or way of scavenging CO_2_ from some other unknown mechanism. To corroborate that the Mixed population was not missing contig sequences that may indicate growth on formate, we looked for unbinned contigs encoding for formate dehydrogenase that may belong to *Methanobacterium*. While many formate dehydrogenases were identified, results from Blastp searches indicated the homology of these genes were not related to any *Methanobacterium* strains (Altschul et al., 1990). Therefore, Mixed *Methanobacterium* populations are demonstrating an alternative method to cope with DIC limitation while also being able to maintain an osmotic balance in the high pH fluids.

Acetate would seem a viable carbon source to support methanogenesis within the hyperalkaline conditions of fluids within the Samail Ophiolite. A study modeling the free energy yield of different substrates in the Santa Elena Ophiolite in Costa Rica demonstrated when acetate comprises a greater proportion of the dissolved organic carbon (DOC) concentration, then the free energy yield becomes more favorable to methanogens (Crespo-Medina et al., 2017). Thermodynamic modeling of biological methanogenesis reactions within the Samail Ophiolite indicates acetoclastic methanogenesis as the most favorable in hyperalkaline conditions (Nothaft et al., 2021b). Given the concentration of acetate is nearly double that of formate while DIC remains incredibly low in the Samail Ophiolite, acetate likely plays a valuable role in the subsurface carbon cycle (Table 1). Yet, the *Methanobacterium* populations here do not display the capacity to assimilate acetate for CH_4_ generation.

It therefore remains puzzling that a metabolic mechanism was not identified within the Mixed population to circumvent limited DIC in the hyperalkaline fluids of borehole BA3A. Given the Mixed population constitutes a significant portion of the relative abundance for the microbial community composition, especially with increasing depth, another yet to be defined adaptation strategy must be at play that cannot be recognized through sequencing data alone. We postulate our hypotheses here as motivation to provide avenues of research benefiting our understanding of *Methanobacterium* populations and biological methanogenesis within the subsurface hyperalkaline system in the Samail Ophiolite. While the Type II population has demonstrated the ability to potentially acquire formate as alternative carbon source, it may be that the Mixed population survives by lowering energetic demands through a sessile lifestyle and increasing interactions with other microorganisms or attaching to a carbon substrate source implied by the enriched presence of glycosyltransferases. The cooperation of other microbiota could result in the oxidation of formate or acetate, analytes which are observed in considerable concentrations within the hyperalkaline fluids compared to other available oxidants. A microbial partner capable of oxidizing formate or acetate may be able to produce a localized source of CO_2_ rapidly consumed by *Methanobacterium*. This collaborative metabolic framework would require a strict spatial proximity of the Mixed *Methanobacterium* population to avoid precipitation of CO_2_ into carbonate minerals in the hyperalkaline conditions.

Acetogens and methanogens have received great attention in serpentinizing systems in efforts to understand early life on Earth, however, the role of additional microbial members in these systems have yet to be explored. Particularly, the ability for specific microorganisms to metabolically cooperate with other members in the community would seem a viable strategy in the nutrient limited hyperalkaline subsurface (Kraus, 2021). Metagenomic sequencing data reveals the presence of the organism *Bellilinea* belonging to the *Anaerolineaceae* family (Supplementary Table 5). *Anaerolineaceae* have been observed in consortia with methanogens in many environments, from anaerobic sludge digesters to marine sediments within Antarctica (Liang et al., 2015; Carr et al., 2018; Dyksma et al., 2020). *Anaerolineaceae* have been observed to degrade n-alkanes and subsequently produce acetate, then successively oxidize acetate into CO_2_ through syntrophic cooperation to support methanogenesis (Callaghan, 2013; Liang et al., 2015). Future work to unravel the role of this organism in potentially aiding the Mixed *Methanobacterium* population and its capability for potential alkane degradation warrant further physiological and sequencing based studies. Whether through the cooperation of syntrophic activity or another metabolic mechanism, further research necessitates additional investigation into how Mixed *Methanobacterium* populations are circumventing DIC limitation in the hyperalkaline subsurface fluids. Future work should consider focusing on cultivated isolates from this system to support the role for niche differentiation and other adaptation strategies of *Methanobacterium* within the subsurface serpentinized fluids of the Samail Ophiolite.

## Conclusion

Metagenomic sequences were collected from subsurface fluids contrasting various geochemical conditions and depths within the Samail Ophiolite, Sultanate of Oman, and allowed the reconstruction of *Methanobacterium* MAGs to investigate niche differentiation within high pH and DIC limitations resulting from hyperalkaline waters. Metapangenomic analysis determined the presence of three distinct *Methanobacterium* populations, where two inhabited the most hyperalkaline pH fluids sampled to date at the Samail Ophiolite. Core genome analysis revealed all *Methanobacterium* populations contain genes indicating DNA scavenging techniques may be a viable strategy to overcome nutrient limitation within the subsurface hyperalkaline, reduced fluids. Metabolic reconstruction corroborated the presence of formate dehydrogenase in Type II populations suggesting formate oxidation to generate intracellular CO_2_ to overcome DIC limitation in hyperalkaline conditions. Further, the accessory genome and functional enrichment of genes unique to the Type II population highlighted proteins relevant to defense against antimicrobials, and the potential use of taurine as an osmoprotectant and source of sulfur. The Mixed population existing in the most hyperalkaline fluids revealed an accessory genome reflecting many cell membrane maintenance mechanisms. The accessory genome of the Mixed population revealed an abundance of various DNA repair genes that likely result from the pressures of osmotic stress on the cell. Additionally, lipoproteins and their associated transporters, as well as glycosyltransferases indicate greater potential for opportunities to interact with microbial biofilms or abiotic surfaces implying a more sessile lifestyle. The significant gene copy number of the *rfaB* glycosyltransferase highlights the need for future research to unravel how this methanogenic population may interact with other microorganisms or adopt another yet to be defined mechanism to overcome DIC limitation, despite a clear metabolic strategy to acquire CO_2_ in the hyperalkaline waters.

The data reported here also highlights the benefits of a metapangenomic approach, and how it is capable of identifying unique properties of microbial members across diverse environmental conditions. This approach distinguished unique adaptations and strategies from *Methanobacterium* populations within the Samail Ophiolite, and would provide an insightful approach to deconvolute how other members may circumvent serpentinization impacted conditions. This work aids our ability to partition niche lifestyles of methanogens in this system to better develop methods for understanding life in other serpentinizing environments on Earth and other planetary bodies.

## Data availability statement

The Methanobacterium MAG assemblies created as a part of this study are accessible under the NCBI Bioproject Number PRJNA930444. The metagenomic sequencing data is available at the JGI IMG database under accession numbers: 3300045950, 3300045482, 3300045454, 3300045456, 3300045455. A reproducible methods document and additional files can be found on Github and includes the code used for this analysis (https://github.com/pthieringer/Metapangenomes_Oman2020).

## Author contributions

PT wrote the manuscript along with help from EB, AT, and JS and conducted all bioinformatic analyses. PT, AT, and JS collected samples and designed the experiment. All authors contributed to the article and approved the submitted version.

## Funding

This work was supported by the National Science Foundation Graduate Research Fellowship (grant no. 1646713): fellow identification number 2018254777 (PT). This work was additionally supported by the NASA Astrobiology Institute “Rock-Powered Life” NAI (NNA15BB02A). The funding agencies did not play a role in design of the study, data collection, or decision to submit the manuscript for publication.

## Conflict of interest

The authors declare that the research was conducted in the absence of any commercial or financial relationships that could be construed as a potential conflict of interest.

## Publisher’s note

All claims expressed in this article are solely those of the authors and do not necessarily represent those of their affiliated organizations, or those of the publisher, the editors and the reviewers. Any product that may be evaluated in this article, or claim that may be made by its manufacturer, is not guaranteed or endorsed by the publisher.
