# Supplementary figures and images for: Metapangenomic investigation provides insight into niche differentiation of methanogenic populations from the subsurface serpentinizing environment, Samail Ophiolite, Oman
Source: Front Microbiol. 2023 Jul 3;14:1205558. doi: 10.3389/fmicb.2023.1205558 (PMC10350532; doi:10.3389/fmicb.2023.1205558)

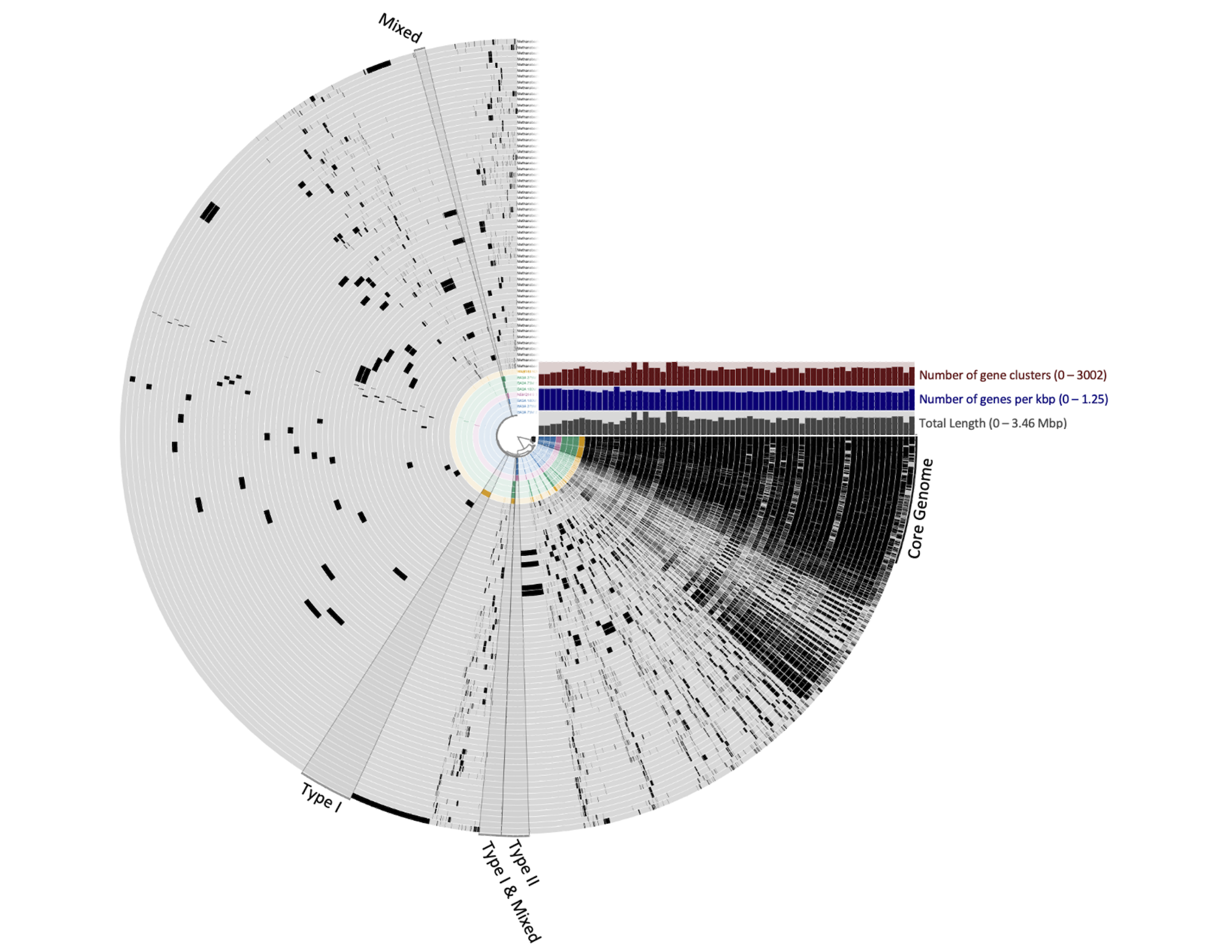

Supplement: Supplementary file 2 [file Image_1.TIFF]
